# Degradation of the cancer genomic DNA deaminase APOBEC3B by SIV Vif

**DOI:** 10.18632/oncotarget.5483

**Published:** 2015-10-31

**Authors:** Allison M. Land, Jiayi Wang, Emily K. Law, Ryan Aberle, Andrea Kirmaier, Annabel Krupp, Welkin E. Johnson, Reuben S. Harris

**Affiliations:** ^1^ Department of Biochemistry, Molecular Biology and Biophysics, Institute for Molecular Virology, Masonic Cancer Center, and Center for Genome Engineering, University of Minnesota, Minneapolis, Minnesota, USA; ^2^ Present address: Department of Biological Sciences, Minnesota State University Mankato, Mankato, Minnesota, USA; ^3^ Department of Biology, Boston College, Boston, Massachusetts, USA; ^4^ Present address: Biogen Idec, Cambridge, Massachusetts, USA

**Keywords:** APOBEC3B, cancer mutagenesis, endogenous DNA deamination, lentiviral Vif, tumor evolution

## Abstract

APOBEC3B is a newly identified source of mutation in many cancers, including breast, head/neck, lung, bladder, cervical, and ovarian. APOBEC3B is a member of the APOBEC3 family of enzymes that deaminate DNA cytosine to produce the pro-mutagenic lesion, uracil. Several APOBEC3 family members function to restrict virus replication. For instance, APOBEC3D, APOBEC3F, APOBEC3G, and APOBEC3H combine to restrict HIV-1 in human lymphocytes. HIV-1 counteracts these APOBEC3s with the viral protein Vif, which targets the relevant APOBEC3s for proteasomal degradation. While APOBEC3B does not restrict HIV-1 and is not targeted by HIV-1 Vif in CD4-positive T cells, we asked whether related lentiviral Vif proteins could degrade APOBEC3B. Interestingly, several SIV Vif proteins are capable of promoting APOBEC3B degradation, with SIVmac239 Vif proving the most potent. This likely occurs through the canonical polyubiquitination mechanism as APOBEC3B protein levels are restored by MG132 treatment and by altering a conserved E3 ligase-binding motif. We further show that SIVmac239 Vif can prevent APOBEC3B mediated geno/cytotoxicity and degrade endogenous APOBEC3B in several cancer cell lines. Our data indicate that the APOBEC3B degradation potential of SIV Vif is an effective tool for neutralizing the cancer genomic DNA deaminase APOBEC3B. Further optimization of this natural APOBEC3 antagonist may benefit cancer therapy.

## INTRODUCTION

The DNA cytosine deaminase APOBEC3B (A3B) was identified recently as a major source of mutation in cancer [1–11]. A3B was initially determined to be upregulated in breast tumors, and this upregulation correlates with increased mutation loads at its preferred DNA deamination motif (*i.e*. 5′-TC-3′) [1]. These mutations have been observed to occur in clusters, termed kataegis, and correlated with translocations and other chromosomal aberrations [6, 12, 13]. Since these findings, A3B has been further implicated in contributing to the mutational load in breast cancer and other malignancies, including bladder, cervical, head/neck, lung, and ovarian cancers [2–11]. Furthermore, clinical data have begun to accumulate, demonstrating that elevated A3B expression correlates with poor outcomes in breast cancer patients [14, 15]. Together, these studies support a model in which A3B is a major source of mutation in cancer that drives tumor evolution, therapy resistance, and poor patient outcomes (reviewed in [16–18]).

A3B is part of the seven-membered APOBEC3 family of proteins, which share the ability to deaminate DNA cytosine to uracil – a pro-mutagenic base. The physiologic roles of the family members are antiviral immunity. However, each APOBEC3 appears specialized to restrict certain pathogens. For example, four members; APOBEC3D (A3D), APOBEC3F (A3F), APOBEC3G (A3G), and APOBEC3H (A3H), have the ability to restrict the lentivirus human immunodeficiency virus-1 (HIV-1) in T lymphocytes by catalyzing mutations in the viral genome and interfering with reverse transcription (many labs, reviewed in [19, 20]). HIV-1 counteracts restriction by binding these APOBEC3 enzymes with the virally encoded Vif protein, and targeting them for E3 ubiquitin ligase-mediated proteasomal degradation (reviewed in [19, 20]). The human A3B (huA3B) protein, however, does not restrict HIV-1 in T cells, and is not neutralized by HIV-1 Vif [21–28].

Most lentiviruses in addition to HIV-1 encode a Vif protein, including simian immunodeficiency virus (SIV), bovine immunodeficiency virus (BIV), feline immunodeficiency virus (FIV), and maedi-visna virus (MVV). The Vif proteins from these viruses also function to degrade the cognate restrictive APOBEC3 proteins from each mammalian host [22, 29–32]. The number and type of APOBEC3 proteins that are encoded by each animal host can vary, with simians expressing seven APOBEC3 proteins (similar to humans), cats expressing five APOBEC3 proteins, and cows and sheep each expressing four APOBEC3 proteins [33–35]. It is generally believed that each Vif protein has undergone evolutionary optimization to specifically degrade the APOBEC3s of each host. However, cross-species degradation has been documented and indeed likely occurs to allow zoonotic transmission [32, 35, 36]. Based on this rationale, we hypothesized that at least one naturally occurring lentiviral Vif would have human A3B (huA3B) antagonizing activity.

To test this idea, we surveyed a panel of Vif proteins from diverse lentiviruses (see Methods for a full listing of viral isolates) and found that SIVmac239 Vif is a potent neutralizer of huA3B, while several other SIV Vif proteins are also capable of promoting huA3B degradation. MG132 treatment inhibited degradation, as did altering the conserved E3 ligase-binding motif, indicating that the degradation likely occurs through the established polyubiquitination mechanism. Finally, we demonstrated that SIVmac239 Vif can prevent huA3B mediated geno/cytotoxicity and degrade endogenous huA3B in multiple human cancer cell lines. Our studies thereby establish SIVmac239 Vif as a molecular tool that may be further developed into a therapeutic strategy to counteract huA3B, decrease tumor mutation rates, and improve patient outcomes.

## RESULTS

### SIVmac239 Vif triggers huA3B degradation

**Figure 1:**
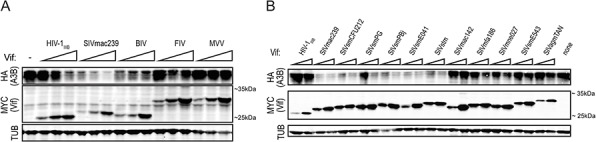
SIVmac239 Vif efficiently degrades huA3B **A.** Immunoblot demonstrating the varying abilities of the lentiviral Vif proteins to degrade huA3B. The lysates were blotted for MYC to detect Vif, HA to detect huA3B, and tubulin (TUB) as a loading control. **B.** Representative immunoblot demonstrating the abilities of Vif from HIV-1_IIIB_ and the indicated SIV isolates to degrade huA3B. The lysates were blotted for MYC to detect Vif, HA to detect huA3B, and tubulin (TUB) as a loading control. The migration positions of molecular weight standards are indicated next to the anti-MYC (Vif) panels.

We and others have previously demonstrated that HIV-1_IIIB_ Vif does not efficiently mediate degradation of huA3B during viral infection [21–25, 37]. To determine huA3B sensitivity to degradation by various lentiviral Vif proteins, we tested the ability of a panel of Vif constructs derived from HIV-1_IIIB_, SIVmac239, BIV, MVV, and FIV to mediate degradation of huA3B. These Vif constructs were transfected into 293T cells at near-equivalent levels based on immunoblots, along with a constant amount of huA3B or vector control (Fig. 1A). The expected sizes of these Vif proteins range from approximately 23.9 kDa for HIV-1_IIIB_ Vif to 30.4 kDa for FIV Vif including the 1.2 kDa carboxy-terminal MYC epitope tag. HIV-1_IIIB_ Vif demonstrated inefficient counteraction of huA3B, as it was only able to mediate degradation of huA3B at the highest expression levels. SIVmac239 Vif was the most efficient at mediating degradation of huA3B, with the lowest level of SIVmac239 Vif mediating a similar level of huA3B degradation as the highest level of HIV-1_IIIB_ Vif. Furthermore, the highest level of SIVmac239 Vif rendered huA3B barely detectable by immunoblot (Fig. 1A). HuA3B cotransfected with BIV Vif showed moderately lower levels of expression regardless of the amount of BIV Vif co-transfected. FIV Vif and MVV Vif did not have any effect on huA3B, regardless of expression levels.

To determine if degradation of huA3B is a property elicited by Vif from multiple SIV strains, we tested a diverse panel of SIV Vif expression constructs. The panel consisted of lentiviruses that naturally infect sooty mangabeys, rhesus macaques, stump-tailed macaques, pig-tailed macaques, cynomolgus macaques, and African green monkeys [38]. As described above, these Vif constructs were expressed in 293T cells at near-equivalent levels based on immunoblots, with huA3B or vector control, and cellular lysates were probed to assess the level of Vif-mediated degradation of huA3B (Fig. 1B). Many of the different SIV Vif proteins caused degradation of huA3B. These include Vif from SIVmac239, SIVsmCFU212, SIVsmPG, SIVsmPBj, SIVsmE041, and SIVstm. Additionally, many of the different SIV Vif proteins were unable to mediate degradation of huA3B. These Vif proteins include SIVmac142, SIVmfa186, SIVmne027, SIVsmE543, SIVagmTAN, as well as Vif from HIV-1_IIIB_ (Fig. 1B). Over multiple independent experiments, SIVmac239 Vif consistently expressed well and elicited a strong huA3B degradation phenotype. For these reasons, additional experiments focused on SIVmac239 Vif.

### SIVmac239 Vif degrades huA3B in a manner analogous to HIV-1_IIIB_ Vif degradation of huA3G

**Figure 2:**
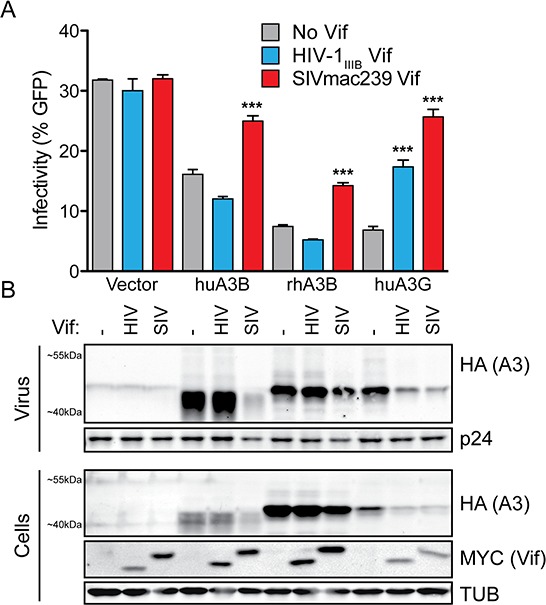
SIVmac239 Vif efficiently counteracts huA3B-mediated restriction of HIV-1 **A.** Bar graph depicting the infectivity (measured as % infected CEM-GFP reporter) of Vif-deficient HIV-1_IIIB_ complemented with vector (grey bars), HIV-1_IIIB_ Vif (blue bars), or SIVmac239 Vif (red bars); produced in the presence of vector control, huA3B, rhA3B or huA3G (*n* = 3; mean and SD shown). Asterisks indicate level of significance, compared to the no Vif condition (***p < 0.001, as determined by one-way ANOVA). **B.** Representative immunoblots for each infection condition are shown beneath each histogram bar. Purified viral particles were blotted for HA to detect A3 and for p24 (Gag) as a loading control. Producer cell lysates were blotted for HA to detect A3, for MYC to detect Vif, and for Tubulin (TUB) as a loading control. The migration positions of molecular weight standards are indicated next to the anti-MYC (Vif) panels.

To determine if SIVmac239 Vif mediates degradation of huA3B in an analogous manner to HIV-1_IIIB_ Vif degradation of huA3G, which has been studied extensively (reviewed in [19, 20]), we tested for Vif-mediated relief of HIV-1 restriction in single-cycle infectivity assays. Additionally, we examined rhesus macaque A3B (rhA3B) susceptibility to SIVmac239 Vif, as this protein is the cognate target of SIVmac239 Vif. HuA3B, rhA3B, huA3G, and vector control constructs were transfected into the 293T cell line with Vif-deficient full-length molecular clone HIV-1_IIIB_. Another vector control, HIV-1_IIIB_ Vif, or SIVmac239 Vif were co-transfected into the cells on separate expression vectors. As shown previously, huA3G restricted viral infectivity in the absence of any Vif protein, but lesser so when HIV-1_IIIB_ Vif was present (Fig. 2A). The ability of huA3G to restrict HIV replication was even more strongly counteracted by SIVmac239 Vif, as reported [39, 40]. Overall, both HIV-1_IIIB_ and SIVmac239 Vif proteins have the capacity to degrade huA3G (Fig. 2B). In contrast, huA3B restricted HIV-1_IIIB_ infectivity both in the absence of Vif protein and in the presence of HIV-1_IIIB_ Vif. Only SIVmac239 Vif was able to relieve huA3B-mediated restriction of HIV-1_IIIB_ (Fig. 2A), and only SIVmac239 Vif promoted degradation of huA3B (Fig. 2B). The rhA3B protein showed a similar restriction profile to huA3B. RhA3B was restrictive in the absence of Vif, and in the presence of HIV-1_IIIB_ Vif. SIVmac239 Vif moderately restored viral infectivity in the presence of rhA3B (Fig. 2A), and had a minor effect on rhA3B degradation (Fig. 2B), as reported [22, 32].

**Figure 3:**
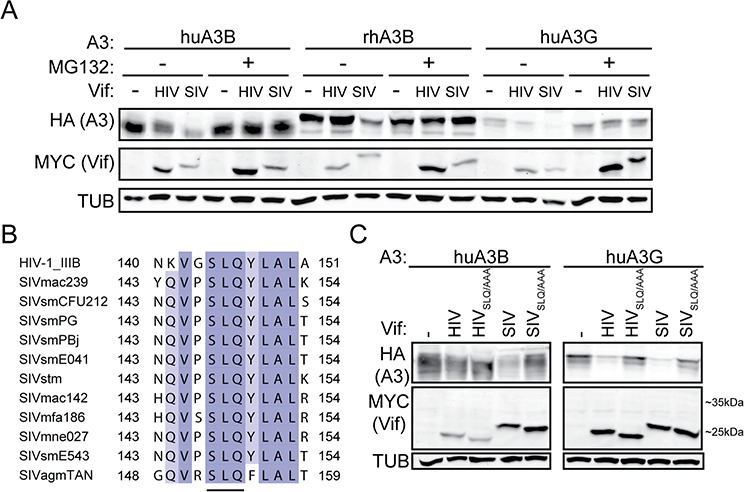
SIVmac239 Vif degradation of huA3B is analogous to HIV-1IIIB Vif degradation of huA3G **A.** Immunoblots demonstrating inhibition of Vif-mediated degradation of A3 proteins in the presence of MG132 (5 μM, 16 hours) or an equivalent amount of acetonitrile as a vehicle control. **B.** Amino acid alignment of the ELOC-binding SLQ region of the HIV-1 and SIV Vif proteins used in this study. The residues are shaded for conservation, with darker shades corresponding to more conserved positions. The residue positions included in the alignment are indicated. The conserved SLQ tri-residue motif is underlined. **C.** Immunoblots demonstrating that SIVmac239 Vif-mediated degradation of huA3B is dependent on the SLQ motif, as is HIV-1_IIIB_ and SIVmac239 degradation of huA3G. Cell lysates were blotted for MYC to detect Vif, for HA to detect A3, and for tubulin (TUB) as a loading control.

To further characterize similarities between huA3G, huA3B, and rhA3B counteraction by HIV-1_IIIB_ Vif and SIVmac239 Vif, we tested whether the observed degradation occurs through a ubiquitin-mediated proteasomal degradation pathway, as is the case for HIV-1 Vif-mediated degradation of huA3G, by inhibiting proteasomal degradation with the compound MG132 [41, 42]. As expected, MG132 inhibited degradation of huA3G by HIV-1_IIIB_ Vif and SIVmac239 Vif (Fig. 3A). SIVmac239 Vif, but not HIV-1_IIIB_ Vif, mediated degradation of huA3B, and this degradation was also inhibited by MG132. RhA3B was somewhat degraded in the presence of SIVmac239 Vif, while HIV-1_IIIB_ Vif was not observed to mediate degradation of rhA3B. Inhibition of the proteasome with MG132 decreased SIVmac239 Vif mediated degradation of rhA3B (Fig. 3A).

We next asked whether the SLQ motif of Vif, which mediates interaction with ELOC of the E3 ubiquitin ligase complex, is important for mediating degradation of huA3B. HIV-1_IIIB_ Vif and SIVmac239 Vif are only 30% identical at the amino acid level, but the SLQ motif is conserved (Fig. 3B). We transfected cells with APOBEC3 and Vif constructs as shown in Fig. 3C. Mutation of the SLQ region to AAA in HIV-1_IIIB_ Vif abrogated its ability to mediate degradation of huA3G. Similarly, mutation of the SLQ region to AAA in SIVmac239 Vif also abolished degradation of huA3G (Fig. 3C). Neither wild-type nor SLQ- > AAA versions of HIV-1_IIIB_ Vif caused degradation of huA3B. The SLQ- > AAA mutation in SIVmac239 Vif prevented Vif-mediated degradation of huA3B, indicating that this Vif protein interacts with the E3 ligase complex to degrade huA3B in a manner similar to the interaction of HIV-1 Vif and huA3G (Fig. 3C).

### SIVmac239 Vif rescues cells from huA3B-mediated cytotoxicity

**Figure 4:**
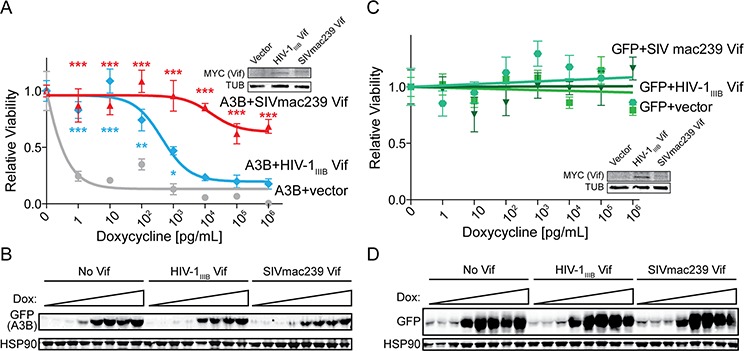
SIVmac239 Vif rescues cells from huA3B-mediated DNA damage and cytotoxicity **A.** Clonogenic assay for T-REx 293 cells expressing huA3B-eGFP with doxycycline induction, and stably expressing vector (grey), HIV-1_IIIB_ Vif (blue), or SIVmac239 Vif (red). Relative viability indicates the ratio of clones that grew in increasing doxycycline, compared to no doxycycline induction (*n* = 3, mean and SD shown). The lysates (inset) were blotted for MYC to detect Vif and tubulin (TUB) as a loading control. Asterisks indicate level of significance, compared to vector condition (*p < 0.05; **p < 0.01; ***p < 0.001, as determined by two-way ANOVA). **B.** Representative immunoblots for cells at each doxycycline (dox) concentration show induction of huA3B in the presence of the indicated Vif constructs. Cell lysates were blotted for GFP to detect huA3B-eGFP and for HSP90 as a loading control. **C.** Clonogenic assay for T-REx 293 cells expressing eGFP with doxycycline induction, and stably expressing vector (squares), HIV-1_IIIB_ Vif (triangles), or SIVmac239 Vif (circles), as described above (*n* = 3, mean and SD shown). The lysates (inset) were blotted for MYC to detect Vif and for tubulin (TUB) as a loading control. **D.** Representative immunoblots for cells at each doxycycline (dox) concentration show induction of GFP in the presence of the indicated Vif constructs. The lysates were blotted for GFP and for HSP90 as a loading control.

HuA3B is geno/cytotoxic in cell culture systems when overexpressed [1, 13, 43]. HuA3B localizes to the nucleus of cells, where it accesses genomic DNA and causes massive amounts of C-to-U deamination events. This leads to abasic sites, catastrophic levels of mutation, and ultimately cell death [1]. To determine if SIVmac239 Vif could save cells from huA3B-mediated cytotoxicity, we stably expressed huA3B-eGFP or eGFP alone under the control of a doxycycline-inducible promoter in T-REx 293 cells, allowing for titratable expression of the protein [1]. Vector control, HIV-1_IIIB_ Vif, and SIVmac239 Vif were expressed stably in the inducible huA3B and GFP cells, and expression was confirmed by immunoblotting (inset images, Fig. 4A & 4B). These cells were plated in increasing concentrations of doxycycline to assess viability in the constitutive presence of Vif and the inducible presence of huA3B.

The cells that inducibly expressed huA3B alone (*i.e.* huA3B + vector control) showed a marked decrease in viability, correlating with increased huA3B expression, with an IC_50_ value of 5 × 10^−1^ pg/mL doxycycline (Fig. 4A). Stable expression of HIV-1_IIIB_ Vif counteracted huA3B at an intermediate level, as demonstrated by significantly increased viability at 1 – 10^3^ pg/mL doxycycline, and increasing the IC_50_ value to 4.4 × 10^3^ pg/mL doxycycline. Additionally, as seen in immunoblots, there is a modest decrease in detected huA3B compared with no Vif (Fig. 4B). Stable expression of SIVmac239 Vif robustly counteracted huA3B and showed fully rescued or significantly increased levels of viability at all tested doxycycline concentrations. The maximal decrease in viability observed was only 30%, preventing the determination of an IC_50_ value. The amount of huA3B detectable by immunoblot is only moderately less than that with no Vif, or HIV-1_IIIB_, and still clearly detectable. These data indicate that SIVmac239 Vif may counteract huA3B through both a canonical degradation mechanism (likely the major pathway based on the aforementioned results) as well as, we speculate, a non-canonical mechanism such as cytoplasmic sequestration. This theory is not without precedent as HIV-1 Vif has been reported to alter the subcellular localization of APOBEC3 enzymes [37]. For comparison, cells that inducibly expressed GFP showed a constant level of viability with increasing GFP expression, regardless of co-expression of HIV-1_IIIB_ Vif or SIV Vif (Fig. 4C & 4D).

### SIVmac239 Vif degrades endogenous huA3B

**Figure 5:**
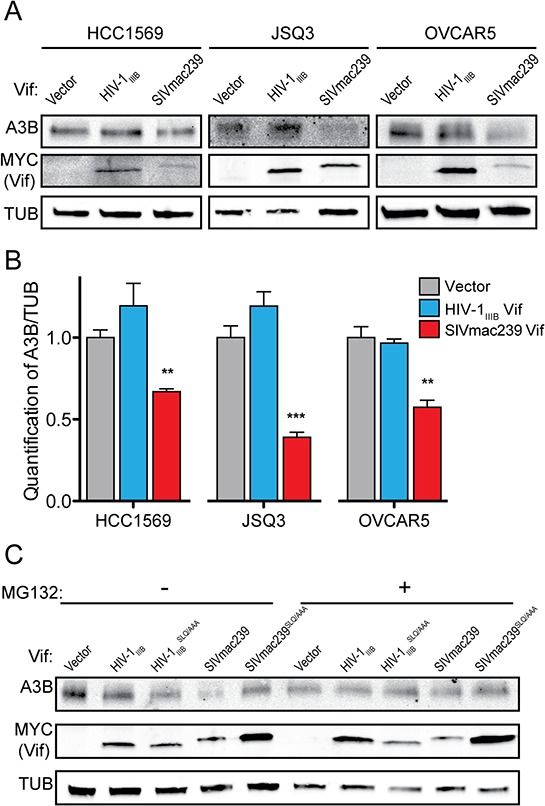
SIVmac239 Vif degrades endogenous huA3B in cancer cells **A.** Representative immunoblots of HCC1569, JSQ3, and OVCAR5 cells expressing empty vector, HIV-1_IIIB_ Vif, or SIVmac239 Vif. Cell lysates were blotted for endogenous huA3B, for MYC to detect Vif, and for tubulin (TUB) as a loading control. **B.** Quantification of endogenous huA3B in cancer cells expressing empty vector, HIV-1_IIIB_ Vif, or SIVmac239 Vif. A3B levels were normalized to Tubulin, and the amount of A3B in the presence of vector, for each cell line, was set at 1 (*n* = 3, mean and SD shown). Asterisks indicate level of significance, compared to vector control (**p < 0.01; ***p < 0.001, as determined by two-way ANOVA). **C.** Representative immunoblot of OVCAR5 cells demonstrating that SIVmac239 Vif-mediated degradation of endogenous huA3B is dependent on the SLQ motif and that this degradation is inhibited in the presence of MG132 (5 μM, 16 hours). Cell lysates were blotted for endogenous huA3B, for MYC to detect Vif, and for tubulin (TUB) as a loading control.

To begin to assess the feasibility of using SIVmac239 Vif or a derivative to counteract endogenous huA3B as an anti-cancer therapeutic, we examined the effect of SIVmac239 Vif in three cancer cell lines that endogenously express high levels of huA3B: HCC1569 cells, a human breast cancer cell line; JSQ3, a human head and neck cancer cell line; and OVCAR5, a human ovarian cancer cell line [1, 3]. These three cell lines were transfected with HIV-1_IIIB_ Vif and SIVmac239 Vif expression constructs, as well as empty vector. No significant difference in levels of endogenous huA3B was observed in cells stably expressing the vector control or HIV-1_IIIB_ Vif (Fig. 5A & 5B). In contrast, HCC1569, JSQ3, and OVCAR5 cells engineered to express SIVmac239 Vif all showed significantly lower levels of huA3B, indicating that SIVmac239 Vif is capable of mediating the degradation of endogenous huA3B in cancer cells (Fig. 5A & 5B).

We further characterized SIVmac239 Vif-mediated degradation of endogenous huA3B in the OVCAR5 cell line. As shown with overexpressed huA3B, mutation of the SLQ region to AAA in SIVmac239 Vif abrogated its ability to mediate degradation of endogenous huA3B (Fig. 5C). Changing the SLQ region to AAA in HIV-1_IIIB_ Vif had no effect, as neither of these proteins mediated degradation of huA3B (Fig. 5C). In further agreement with the overexpression experiments, treatment of the OVCAR5 cells with the proteasomal inhibitor MG132 rescued endogenous huA3B from SIVmac239 Vif-mediated degradation (Fig. 5C). Based on the established mechanism for Vif function, these data indicate that SIVmac239 Vif interacts with ELOC of the E3 ubiquitin ligase complex via its SLQ motif to mediate proteasomal degradation of endogenous huA3B.

## DISCUSSION

A3B contributes to genomic mutation in breast and other cancers, and associates with poor patient outcomes (reviewed in [16–18]). As such, it would be beneficial to cancer research and patient therapy to be able to counteract this potent DNA mutator. A3B is part of a larger family of APOBEC3 proteins, some of which have physiologic functions in restricting HIV-1 replication, and these are counteracted by HIV-1 Vif (reviewed in [19, 20]). HuA3B is not efficiently degraded by HIV-1 Vif [21–28], however, HIV-1 Vif may bind huA3B as indicated by co-immunoprecipitation studies [21, 37]. We hypothesized that the Vif protein from another lentivirus may be capable of counteracting huA3B, as diverse lentiviruses are restricted by differing subsets of the host's APOBEC3 proteins. By surveying a panel of Vif proteins from lentiviruses that infect different animal hosts, we identified SIVmac239 Vif as a potent inhibitor of both overexpressed and endogenously upregulated huA3B, degrading huA3B in an analogous manner to HIV-1_IIIB_ Vif mediating degradation of huA3G.

In testing the conservation of the ability of Vif proteins from various SIV strains to degrade huA3B, we found that many but not all SIV Vif proteins share this trait. Such an ability to degrade huA3B is potentially due to structural and functional conservation of huA3B with simian APOBEC3 proteins and the ability of some of these simian enzymes to restrict the relevant SIV strains, thus being legitimate targets of the respective Vif ubiquitin ligase complexes. For example, rhA3B has been demonstrated here and in other studies to be counteracted by SIVmac Vif, suggesting that this protein may be relevant for SIVmac infection of rhesus macaques [22, 32]. In contrast, HIV-1 is not restricted by huA3B in T cells, the major cellular target of HIV-1, and HIV-1 Vif does not efficiently counteract huA3B [21–28]. These species specificities can be utilized to further understand the relevant protein interaction surfaces. For instance, the ability of HIV-1 Vif to counteract huA3F but not rhA3F has led to better understanding to this protein interaction surface [32, 44–46]. By better understanding the interaction between huA3B and SIVmac239 Vif, SIVmac239 Vif may be engineered to become more efficient at degrading huA3B and to increase its specificity for huA3B over other APOBEC3 proteins, resulting in a better molecular probe and cancer therapeutic.

Interestingly, although we clearly showed that SIVmac239 Vif is capable of degrading huA3B, it also appeared capable of neutralizing the cytotoxic effects of huA3B beyond the canonical degradation mechanism. Based on prior studies [37], we hypothesize that SIVmac239 Vif may also bind huA3B in the cytosol and prevent it from importing into the nuclear compartment. Such a sequestration mechanism could help protect genomic DNA from huA3B's genotoxic activities, as has been inferred for the related APOBEC3A (A3A) protein [47, 48]. However, this theory needs extensive testing for validation and to distinguish it from other possible mechanisms. HIV-1_IIIB_ Vif was also able to mediate neutralization of huA3B, albeit at a much lower level than SIVmac239 Vif. This may be related to binding rather than degradation of the protein. This observation also suggests that HIV-1 Vif could protect cells from huA3B's oncogenic effects, and that HIV-1 infected individuals may be partly protected from the onset or progression of some malignancies. However, this does not seem to be the case as HIV-1 infection is associated with an increased risk of several AIDS-defining and non-AIDS defining cancers [49, 50].

This study is the first to show that SIVmac239 Vif can degrade the genomic DNA mutator huA3B in living cells. Degradation of endogenous huA3B was observed in cell lines representing three different types of human cancer, suggesting that although cellular factors that regulate huA3B are not yet elucidated, this finding may have implications for treating a wide range of huA3B effected malignancies. The data presented here have strong implications for developing SIVmac239 Vif as a molecular tool for future studies on the mutagenic properties of huA3B, and for neutralizing huA3B in cancer to halt tumor mutagenesis, prevent therapy resistance, and improve the treatment and prognosis of cancer patients.

## MATERIALS AND METHODS

### APOBEC3 expression constructs

The APOBEC3 proteins huA3B (GenBank accession no. NM004900), huA3G (GenBank NM021822), and rhA3B (GenBank JF714485, but with the asparagine at amino acid residue 316 restored to aspartate [51]) were expressed with carboxy-terminal HA tag in the pcDNA3.1(+) vector (Invitrogen). cDNA was provided by Dr. Theodora Hatziioannou (Aaron Diamond AIDS Research Center, New York) [32]. Additionally, huA3B was expressed with a carboxy-terminal eGFP tag in the doxycycline-inducible pcDNA5TO vector (Clontech).

### Vif nomenclature and expression constructs

Each Vif protein is described by virus type (HIV, SIV, etc) and a strain/isolate identifier (IIIB, mac239, etc) according to standard conventions in the retrovirus field. The lentiviral Vif proteins from HIV-1_IIIB_ (protein sequence matches GenBank EU541617), SIVmac239 (GenBank AY588946), BIV_BIM127_ (GenBank M32690), MVV_1514_ (GenBank M60610), and FIV_NSCU_ (GenBank m25381) were codon optimized (GenScript Corporation) and expressed with a carboxy-terminal MYC tag in the pVR1012 vector [29]. Vif expression constructs from SIVsmCFU212 (GenBank JX860407), SIVsmPG (GenBank AAC68657), SIVsmPBj (GenBank AAB22996), SIVsmE041 (GenBank HM059825), SIVstm (GenBank AAA91941), SIVmac142 (GenBank Y00277), SIVmfa186 (GenBank KF030930), SIVmne027 (GenBank U70412), SIVsmE543–3 (GenBank U72748), and SIVagmTAN (GenBank AAC57053) were derived originally in the Johnson lab (Boston College) [38]. These cDNAs were subcloned into the pVR1012 vector with a carboxy-terminal MYC tag. The Vif expression construct pVR1012 was a generous gift of Dr. Xiao-Fang Yu (John Hopkins, Baltimore). For transient expression in HCC1569 and JSQ3 cells, the constructs were transfected with TransIT-2020 (Mirus) and TransIT-X2 (Mirus), respectively. For stable expression in OVCAR5 cells, HIV-1_IIIB_ and SIVmac239 Vif were subcloned into the pLenti4-Hygro-TO backbone, transduced into OVCAR5 cells, and a stably expressing pool was selected with hygromycin.

### HIV constructs

The Vif proficient and Vif deficient (X26X27) HIV-1_IIIB_ A200C proviral expression constructs (GenBank EU541617) have been reported [52].

### Cell lines

293T cells, T-REx 293 (Invitrogen) cells, and JSQ3 cells were maintained in Dulbecco's modified Eagle medium (DMEM) containing 10% fetal bovine serum (FBS) and 0.5% penicillin-streptomycin (P/S). CEM-GFP, HCC1569, and OVCAR5 cells were maintained in RPMI medium with 10% FBS and 0.5% P/S. The CEM-GFP HIV reporter cell line was obtained from the NIH AIDS Reagent Program [53]; the breast cancer cell line HCC1569 from ATCC; the head and neck cancer cell line JSQ3 from Dr. Mark Herzberg (University of Minnesota); and the ovarian cancer cell line OVCAR5 from the Mayo Clinic ovarian cell line repository.

### Immunoblotting

Cell lysates were prepared by resuspending washed cell pellets directly in 2.5 X Laemmli sample buffer. Viral particles were purified from the filtered supernatant by centrifugation prior to resuspension in 2.5X Laemmli sample buffer. A3-HA was detected with monoclonal mouse anti-HA (BioLegend), Vif-MYC was detected with polyclonal rabbit anti-MYC (Sigma-Aldrich), Tubulin (TUB) was detected with monoclonal mouse anti-α-Tubulin (Covance), HIV-1 Gag was detected with monoclonal mouse anti-HIV-1 p24 (NIH AIDS Reagent Program) [54], A3-GFP was detected with monoclonal mouse anti-GFP (Clontech), HSP90 was detected with mouse anti-HSP90 (BD Biosciences). A3B was detected with rabbit monoclonal anti-A3B [55] (Brown *et al*., *in prep*). To determine endogenous huA3B degradation, the huA3B and Tubulin bands were quantified from immunoblots using ImageJ (1.42q), and huA3B levels were normalized to those of Tubulin. These values were analyzed using a two-way ANOVA. Bonferroni's method for post-hoc testing was used to compare the amount of huA3B in the presence of vector, HIV-1_IIIB_ Vif, and SIVmac239 Vif. Statistical analyses were done with Prism 5 (GraphPad Software Inc.).

### Vif degradation

293T cells were transfected in triplicate with pVR1020-Vif-MYC or empty vector, at levels normalized by immunoblot, and pcDNA3.1 A3-HA, or empty vector, as indicated, using PEI (polyethyleneimine; Polysciences, Inc.). The following amounts of Vif expression construct were transfected for Fig. 1A: HIV-1_IIIB_ 50–200 ng; SIVmac239 50–200 ng; BIV 100–400 ng; FIV 50–200 ng; MVV 100–400 ng. For Fig. 1B, the following amounts of Vif expression construct were transfected: HIV-1_IIIB_ 50–100 ng; SIVmac239 100–200 ng; SIVsmCFU212 200–400 ng; SIVsmPG 200–400 ng; SIVsmPBj 200–400 ng; SIVsmE041 200–400 ng; SIVstm 400–800 ng; SIVmac142 200–400 ng; SIVmfa186 200–400 ng; SIVmne027 200–400 ng, SIVsmE543 200–400 ng; SIVagmTAN 400–800 ng. After 48 hours, the cells were harvested for immunoblot analysis. To inhibit proteasomal degradation, MG132 (American Peptide) was added at 5 μM, 16 hours before harvesting the cells.

### HIV-1 single cycle infection with replication-proficient virus

The single-cycle infectivity assays were performed as previously reported [22] by transfecting 293T cells (TransIT-LT1; Mirus) in triplicate with 1 μg of a Vif-deficient HIV-1 proviral expression construct along with 25 ng of A3-HA expression construct or empty vector, and 25 ng of Vif-MYC expression construct or empty vector. After 48 hours, purified virus-containing supernatants were used to infect the CEM-GFP HIV-1 reporter cells, and cell and viral particle lysates were prepared for immunoblotting. Infectivity was analyzed using a one-way ANOVA. Dunnett's method for post-hoc testing was used to compare increases in infectivity in the presence of the Vif expression constructs with vector control. Statistical analyses were done with Prism 5 (GraphPad Software Inc.).

### Flow cytometry

HIV-infected CEM-GFP cells were prepared for flow cytometry by fixation in 4% paraformaldehyde. GFP fluorescence was measured on a BD FACS Canto II flow cytometer (BD Biosciences). All data were analyzed using FlowJo flow cytometry analysis software (version 8.8.7). GFP fluorescence was quantified from gated live cell populations.

### Viability assay

T-REx 293 cells (Invitrogen), which stably express the tetracycline repressor, were transfected with pcDNA5TO-A3B-eGFP and pcDNA5TO-eGFP constructs using TransIT-LT1 (Mirus). Stable clones were selected with hygromycin and blasticidin. These T-REx 293 huA3B and T-REx 293 GFP stable clones were further engineered to stably express HIV-1_IIIB_ Vif-myc, SIVmac239 Vif-MYC or vector by transfection of pcDNA3.1 expression constructs and selection with G418. To assess viability, equal numbers of cells were plated in triplicate in increasing doxycycline concentrations and clones were allowed to form. The clones were quantified using ImageJ (1.42q) software. In parallel, these cells were plated in increasing doxycycline concentrations and harvested after 48 hours for immunoblotting. Viability data were analyzed using a two-way ANOVA. Bonferroni's method for post-hoc testing was used to compare viability in the presence of vector control to the viability with HIV-1_IIIB_ Vif and SIVmac239 Vif. Statistical analyses and TCID_50_ were done with Prism 5 (GraphPad Software Inc.).
